# Understanding the Key Determinants of Cardiovascular and Metabolic Disease Progression to Develop Effective Therapeutic Strategies

**DOI:** 10.3390/biom14101281

**Published:** 2024-10-11

**Authors:** Adriana Georgescu

**Affiliations:** Pathophysiology and Pharmacology Department, Institute of Cellular Biology and Pathology “Nicolae Simionescu” of the Romanian Academy, 050568 Bucharest, Romania; adriana.georgescu@icbp.ro

Cardiovascular disease (CVD) is a general term that is used to describe a range of conditions affecting the cardiovascular system. These conditions have a significant impact on the elderly population, with a lesser prevalence among younger individuals. CVDs are the result of metabolic disorders, such as dyslipidaemia and diabetes, which affect the heart and blood vessels. The principal cause of CVDs is atherosclerosis, which can occur in all the arteries of the body. This process results in the formation of atherosclerotic plaques, which are composed of fats, cholesterol, calcium, and other substances and molecules that accumulate in the arterial walls.

With the passage of time, the plaques grow and harden, which results in a reduction in the elasticity and inner diameter of the arteries and a subsequent restriction of blood flow. Eventually, the atherosclerotic plaques may rupture, forming a blood clot (thrombosis) that can result in the further restriction or even the complete blockage of blood flow throughout the body.

The early detection and elucidation of the cellular and molecular mechanisms involved are essential for the prevention, intervention, and monitoring of the progression of CVDs.

This Special Issue entitled “Recent Advances in Cellular and Molecular Mechanisms of Cardiovascular and Metabolic Diseases” comprises eleven original research papers and six reviews by experts in the field of cardiovascular and metabolic diseases. It provides readers with the latest news and advances in relation to these pathological processes. The seventeen scientific papers contribute to our understanding of these complex pathologies and highlight important key elements that could inform the development of effective therapeutic strategies for cardiovascular and metabolic diseases.

The study by Koenen et al. investigated the extracellular vesicle (EV)-mediated signalling process from fatty acid-treated human hepatocytes to hepatic stellate cells. The authors demonstrated that these steatotic hepatocytes are capable of activating myofibroblasts via the EVs they produce, thereby inducing pro-fibrotic responses in cultured stellate cells during metabolic-associated fatty liver disease (MAFLD) [1].

It is widely acknowledged that the overproduction of EVs, coupled with inadequate reabsorption, represents a pivotal mechanism underlying organ fibrosis [2]. The involvement of EVs in the bilateral link between CVD and cancer has also been discussed by Badila et al. [3] in a review included in this Special Issue. In this review, the authors describe important aspects of CVD development in cancer patients and underline the role of EVs as diagnostic biomarkers and drug delivery vehicles in this relationship. The specific place of EVs in the dialogue between CVD and cancer is far from being elucidated, thus stipulating the need for basic, clinical, and translational research. However, there are many studies on the diagnostic, prognostic, and therapeutic tool value of EVs in atherosclerosis and CVDs [4,5,6,7,8].

Interestingly, another experimental study carried out both in vivo and in vitro showed that adenine reduced atherosclerosis in ApoE knockout mice and had atheroprotective effects on RAW 264.7 cells (monocyte/macrophage-like cells) through its effect on cholesterol efflux [9]. In vitro experiments have also highlighted the association between semicarbazide-sensitive amine oxidase (SSAO) and lysyl oxidase (LOX) in rat aortic vascular smooth muscle cells (VSMCs), with LOX being identified as a key regulator of SSAO activity, and VAP-1 protein and Aoc3 mRNA expression in early passage rat aortic VSMCs [10].

Studies in both animal models and patients are of particular importance in this Special Issue. For example, a randomised controlled trial showed that preoperative remote ischaemic preconditioning (RIPC) did not significantly affect the metabolome in patients undergoing vascular surgery [11]. However, the authors concluded that further studies are needed to determine with certainty whether RIPC affects the metabolome and, more importantly, how these metabolomic changes might correlate with markers of cardiac and renal injury [11].

In the study by van der Vaart et al., the authors demonstrated a paradoxical role of circulating ketone bodies (KBs) on glycaemic control in type 2 diabetes (T2D), whereby elevated KB levels are cross-sectionally associated with worse glycaemic control, but with better long-term glycaemic control [12]. Fasting plasma KBs are known to be elevated in patients with T2D and may influence glycaemic and disease progression. This proof-of-concept observational study was based on the hypothesis that fasting plasma KBs may be potential biomarkers for impaired glycaemic control in patients with T2D, but may also be associated with improved long-term metabolic control [12].

Another interesting original paper showed that a period of at least 8 weeks of vigorous exercise was sufficient to induce significant improvements in platelet sensitivity to prostacyclin with and without dual antiplatelet therapy (DAPT; aspirin and a P2Y12 antagonist) in previously sedentary postmenopausal women [13]. This effect appears to be due to the improved function of the platelet prostacyclin receptor and signalling molecules, such as adenylate cyclase or cyclic adenosine monophosphate. The results highlight the importance of promoting physical activity to reduce thrombotic risk in postmenopausal women and suggest that exercise status should be considered when prescribing DAPT [13].

Important studies have highlighted the role of adiponectin, a protein secreted by adipocytes, in metabolic homeostasis and the incidence of CVD. In this regard, the study by Zahradka et al., performed both in vivo and in vitro, showed that globular adiponectin, whose formation is mediated by thrombin, increases adipocyte size and adipose tissue mass, but does not affect fasting glucose levels in obese patients [14].

Metabolic remodelling in patients with different haemodynamic subtypes of severe aortic stenosis (AS) was first investigated by Bengel et al. The results showed clear differences for metabolites belonging to haemoglobin metabolism, diacylglycerols, and dihydrosphingomyelins. However, the authors pointed out that further studies are needed to clarify their pathophysiological significance [15].

Another interesting study was based on the idea that fluctuations in glucose concentrations occur daily (postprandially) in the blood of both normoglycaemic and hyperglycaemic subjects. Thus, in an in vitro study, Toma et al. investigated the effects of oscillating glucose on endothelial cell function and the molecular mechanisms involved [16]. They showed that oscillating glucose induces endothelial cell dysfunction through a mechanism involving the upregulation of Ninjurin-1, a molecule proposed as a new therapeutic target for the alleviation of endothelial cell dysfunction. Other molecules proposed to intervene in this process of endothelial dysfunction as a result of increased oxidative stress and an inflammatory phenomenon are TNFR1, RAGE, Cav-1, SR-B1, VAMP-3, Cav-1, TNFR1, and SR-BI proteins [16].

CVD is a major health problem for populations worldwide, but a recent study published in this Special Issue notes that mortality from CVD is higher in Russia then in other European countries. The aim of this study was to define the prevalence of low-grade systemic inflammation (LGSI) and its associated factors in a Russian population [17]. Thus, abdominal obesity was shown to be strongly associated with LGSI regardless of sex, while the profiles of LGSI correlates differed between the two sexes, suggesting that LGSI development has different sex-specific characteristics and health consequences [17].

It is well established that atherosclerotic disease, regarded as a condition affecting the intima, shares common characteristics with aneurysms, diseases affecting the media. These include matrix remodelling, macrophage homing, and calcification. Aneurysms are most commonly formed at the abdominal aorta, a condition known as abdominal aortic aneurysm (AAA). However, they can also occur in the peripheral area, leading to the formation of aneurysms in the popliteal artery (PAA). In general, patients with AAA also have atherosclerosis. Pauli et al. suggest that PAA may have a distinct pathophysiology compared to AAA. In particular, apolipoprotein E (ApoE) is specifically more highly expressed in PAA tissue and may be involved in vascular smooth muscle cell phenotype rescue [18].

The review by Younes et al. presents novel data on the mechanisms of failed resolution in the context of arrhythmogenic inflammation, fibrosis, and right heart disease [19]. The process of inflammation is one that is extremely complex, involving a number of stages. These stages begin with the initiation and resolution of the inflammatory response, which is aimed at promoting homeostasis. However, if the inflammatory status of the tissue is not resolved, this can result in the generation of chronic inflammatory disorders. This is due to the fact that the structural lesions that are caused by the aggravation of the inflammatory response, as well as the development of a fibrous area, can result in a loss of function. In this review, the authors present a discussion of the paradigm of failed resolution mechanisms that may promote arrhythmogenicity in the inflammatory state induced by right heart disease [19].

Heart failure (HF) is a prevalent condition affecting a significant proportion of the population. Consequently, there has been a notable increase in research activity in this field in recent years. The results of both animal studies (pre-clinical) and clinical studies indicate that there are significant differences between the various subtypes of heart failure (HF), including those with preserved and reduced ejection fraction (HFpEF and HFrEF). One of the reviews published in this Special Issue provides a comprehensive analysis of the latest clinical and preclinical evidence, exploring the full potential of key metabolic alterations in the onset and progression of both HF subtypes [20].

Another significant pathology addressed in the review by Bariani et al. is arrhythmogenic cardiomyopathy (ACM), a genetically predisposed myocardial disease.

ACM is a heart muscle disease that is characterised by the presence of prominent non-ischemic myocardial scarring, which predisposes the heart to ventricular electrical instability [21]. ACM can result in sudden cardiac death, particularly among younger individuals, due to the emergence of ventricular arrhythmias caused by myocyte necrosis with fibrofatty replacement. In order to gain a deeper understanding of the clinical characteristics of patients with ACM presenting with “hot-phase” episodes, the authors conducted a critical evaluation of the relevant literature [22].

A review was conducted to elucidate the cellular and molecular mechanisms underlying cardiopulmonary complications in sickle cell disease (SCD). Additionally, the latest scientific evidence derived from clinical and laboratory studies was highlighted [23]. It has been established that inflammation plays a significant role in the pathogenesis of SCD-associated cardiopulmonary complications. Consequently, the presentation included an overview of inflammatory mediators and potential novel treatments for cardio-pulmonary complications in SCD [23].

The review by Sabatino et al. provides a comprehensive overview of the current understanding of the role of thyroid hormones in the recovery and protection of the brain from disease and injury. The primary factors and mechanisms regulating thyroid hormone effects in cells were elucidated [24]. A substantial body of evidence suggests that thyroid hormones are crucial for optimal brain development and functionality. Moreover, they may play a pivotal role in the processes underlying recovery from neurological disorders and brain injuries. The administration of thyroid hormones (T4, T3, or a combination of the two) to animal models has been demonstrated to enhance neuronal survival and improve neurological outcomes. Nevertheless, this remains an area of research open to further investigation, with the objective of defining the appropriate dose and timing for optimal thyroid hormone therapy. It has been demonstrated that disturbances in thyroid hormones during early life can result in a series of irreversible morphological and biochemical changes. Nevertheless, further research is required to substantiate this hypothesis [25].

In general, the early diagnosis of cardiovascular and metabolic complications through the early recognition and application of molecular risk factors before irreversible organ damage occurs would contribute to an improved quality of life. This is because patients with these complications are now living longer due to the availability of therapies that modify disease.

It is my sincere hope that readers of this Special Issue of the Journal “Biomolecules” will be captivated by the latest news in the field of cardiovascular and metabolic diseases, and especially by the cellular and molecular mechanisms that underlie their occurrence and that help in the development of effective therapies.
